# Assessing Olive Oil Quality Using Different DNA-Based Methods

**DOI:** 10.3390/plants13223220

**Published:** 2024-11-16

**Authors:** Giovanna Moscato, Savino Bonavita, Teresa Maria Rosaria Regina

**Affiliations:** 1 Dipartimento di Biologia, Ecologia e Scienze della Terra (DiBEST), Università della Calabria, Via Ponte P. Bucci, 87036 Arcavacata di Rende, Italy; giovannamoscato1397@gmail.com; 2 Laboratorio Dolciaria Monardo Srl, Località Carromonaco, 89831 Soriano Calabro, Italy; qualita@dolciariamonardo.com

**Keywords:** olive oil, mold fungi, endpoint PCR, real time PCR, LAMP

## Abstract

Olive oil is appreciated worldwide for its unique nutritional and organoleptic properties. It is rich in unsaturated fatty acids and antioxidants, which are well-known for their health benefits. The qualitative characteristics of olive oil can be adversely affected by various biotic and abiotic factors. Particularly, microbial pathogens, such as mold fungi, can cause the deterioration of the oil and, thus, be a serious risk to consumer health. In this study, the effectiveness of DNA-based methods, i.e., endpoint PCR, Real-Time PCR (RT-PCR), and loop-mediated isothermal amplification (LAMP), all based on the ITS2-28S region, were used to evaluate the fungal contamination of samples of extra virgin olive oil. All the DNA techniques were able to detect, albeit at different levels, fungal infections affecting some of the basic quality parameters of the olive oils analyzed. However, compared to endpoint PCR and/or RT-PCR, the LAMP assay greatly simplified and accelerated the identification of pathogenic mold in the oil samples. This may encourage the olive oil industry to adopt this method in order to offer the consumer an oil with specific health parameters and therefore guarantee the safety and quality of this precious food product.

## 1. Introduction

The olive tree, *Olea europaea* L., is one of the most ancient woody horticultural species of the Mediterranean basin, where it is still the most important crop for its edible fruit and its oil.

Obtained mechanically only from the olive fruit, edible olive oil, and especially extra virgin olive oil (EVOO), is considered to be of the highest quality and a healthy food, as it is rich in phenolics, carotenoids, tocopherols, and anthocyanins, which protect against various degenerative and chronic diseases [1,2].

The quality of EVOO varies depending on the variety, environmental conditions, and many other parameters, i.e., agronomic practices, harvest time, olive ripening, and storage of harvested olives [3]. Oil quality is very important in the olive oil industry and marketing. There are a number of chemical indices related to olive oil quality, the most important of which are free fatty acids, peroxide number, p-anisidine value, and spectrophotometric indexes (i.e., specific absorption at K232 nm and K270 nm). Particularly, the free acidity and peroxide value are mainly used to assess oxidative degradation (rancidity) of oils, the latter leading to the development of undesirable flavors and odors that clearly do not meet consumer expectations.

The threshold value for free acidity set by the International Olive Council (IOC) [4] is around 0.80% for EVOO, while the threshold value for the peroxide content has been set at 20 meqO_2_/kg. An oil may be considered not to belong to the category declared if at least one analytical parameter does not comply with the standard indices. An increase in peroxide levels in an oil can be caused by a number of factors, including the prolonged exposure of olives to heat, light, and oxygen prior to oil extraction, or increased damage between harvesting and processing [5].

The presence of dangerous microorganisms such as yeasts, bacteria, and fungi also affect peroxides. Indeed, leaving olives on the ground, in jute bags, or in poorly ventilated stores for some time before processing can create ideal conditions for the growth of fungi and molds, the most representative genera being *Aspergillus* and *Penicillium* [6]. Also, the presence of fungal spores in oil cannot be excluded [7], even though the composition of this liquid product is not favorable to the development of microorganisms because of the low water content and the presence of fat as the only food source.

Several mold fungi are producers of a group of secondary metabolites (mycotoxins), including aflatoxin, alkaloids, fumonisins, and ochratoxin A [8,9,10], which act as genotoxic, mutagenic, and carcinogenic agents and cause other risks to human health [11,12]. The *Aspergillus* and *Penicillium* mold genera, in particular, are considered the most frequent toxigenic fungi in Europe [11]. The maximum level of the different types of aflatoxins in oilseeds has been set by the European Union at 4.0 µg/kg [13,14].

Therefore, not only are olive oil quality indices important, but also the early and accurate detection of pathogens contaminating olive oil is crucial to ensure consumer safety and protection.

Traditional methods for the detection of filamentous fungi include morphological characterization after growth on agar media [15].

Some *Aspergillus* species, such as *A. parasiticus* and *A. sojae*, have been identified by the color of the colonies, the diameter of the conidia, or by the use of a selective medium [16]. However, the morphological differences are not always obvious, and accurate identification is a time-consuming process that requires laboratory facilities and mycological expertise [17,18].

On the other hand, molecular methods, particularly PCR-based procedures, have been widely used for the identification of mycotoxigenic strains, being faster, more sensitive, and more specific tools than culturing techniques [19,20,21]. However, PCR-based methods often require expensive reagents and sophisticated temperature-controlled equipment, which often limit their potential practical use as rapid and inexpensive field tests. Conversely, the loop-mediated isothermal amplification (LAMP) reaction has been described as a cost-effective and easy-to-use method [22]. The high specificity of this technique is based on the use of four to six specific primers that recognize different DNA sequences on a target gene, and a specific DNA polymerase (usually the *Bst* DNA polymerase from *Bacillus stearothermophilus*) with a strand displacement activity, which amplifies them under isothermal conditions (at 60 °C to 65 °C), demonstrating high efficiency and rapidity. Since the amplified products can be easily detected in several ways (i.e., by checking the amplified products on agarose gel electrophoresis, by using colorimetric indicators, or by naked eye observation) [22,23,24], LAMP can be used in the analytical laboratory as well as in the field with small, portable instruments.

Over the past decades, the LAMP assay has demonstrated its usefulness in detecting *Aspergillus* spp. that commonly contaminate samples of rice, nuts, raisins, dried figs, and powdered spices, or in differentiating between aflatoxigenic and non-aflatoxigenic producing *Aspergillus* strains [25,26,27,28,29,30].

In this study, the specificity, sensitivity, efficiency, and speed of the three DNA-based approaches, namely endpoint PCR, real-time PCR (RT-PCR), and LAMP assay, all based on the nuclear region that includes the internal transcribed spacer 2 and the large subunit ribosomal DNA (ITS2-28S) sequence, were compared for the first time to determine which would be most suitable for the rapid monitoring of the potential *Aspergillus* spp. contamination of EVOO samples, collected from different areas of the Calabria region of southern Italy. With an average annual production of 100,000 tons, Calabria is the second-largest olive oil-producing region in Italy, with over 95% of its olive groves dedicated to oil production and the small remainder to table olive production.

The results of our comparative analysis may be of interest to producers, manufacturers, distributors, and retailers, providing a simple diagnostic test for the rapid assessment of the quality of EVOOs.

## 2. Results

### 2.1. Quality Parameters of the Oil Samples

We started our study by collecting 20 samples of mono- and multivarietal EVOO, listed in Table 1, from Calabrian olive mills and supermarkets, which were first used to perform some basic chemical analyses, described in Section 4.

Among the analytical indicators, the amount of free fatty acids, measured as acidity, is certainly the most important parameter for olive oil quality and all our samples had acidity values below the legal limit of 0.8% for EVOO (Table 2). In addition, the peroxide value and p-anisidine value are quality indicators of the degree of oxidation in oil, providing insight into oxidative degradation and estimated shelf life.

The peroxide value, in particular, showed considerable variation and was remarkably high (above the threshold of 20 meqO_2_/kg) in some EVOO samples (Table 2), which cannot thus be considered products of good quality and should not be marketed.

As prolonged exposure of olives to the environment prior to oil extraction, increased damage between harvest and processing, as well as mold contamination are known to have a negative impact on oil peroxide levels, we next decided to test the EVOOs investigated here for potential fungal infection using DNA-based methods.

### 2.2. Olive Oil DNA Isolation

Total genomic DNA from all oil samples was extracted, as described in Section 4 Materials and Methods, and quantified at 260 nm, yielding a minimum concentration of 5 ng/μL from EVOO CAS and a maximum of 20 ng/μL from EVOO MV2 in a final volume of 50 μL. The quality of the DNA, as determined by the ratio 260/280 nm, was similar in all the samples (1.31 ± 0.01). The low absorbance ratios found in the olive oil DNAs were indicative of the presence of protein, phenol, or other contaminants in the samples [31], which are known to interfere with subsequent amplification reactions or cause false positives/negatives.

To verify the recovery of PCR-compatible oil DNAs, aliquots of each EVOO DNA were first used as templates to set up the amplification reactions with the primers for the chloroplast *trn*L intron sequence (Table 3), which represent one of the most popular and widely used primers for investigating plants even with highly degraded target DNA [32].

**Figure 1:**
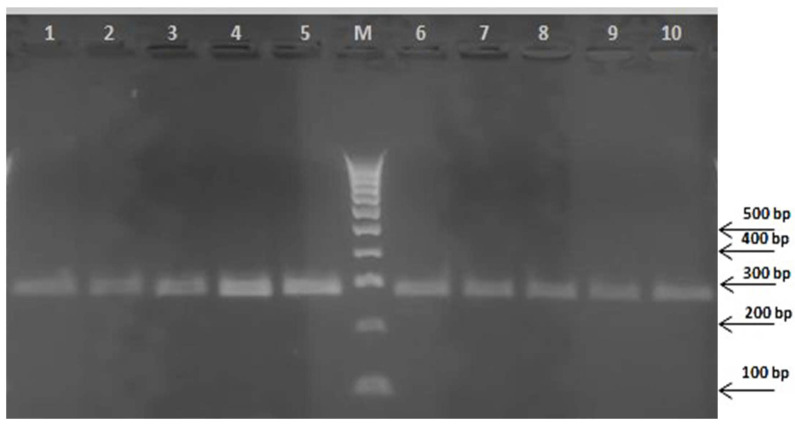
Agarose gel electrophoresis of the *trn*L amplicon obtained by PCR amplification of the genomic DNA from some of the monovarietal and multivarietal olive oils analyzed in this study. Samples: CAS (lane 1), CIC (lane 2), NOC (lane 3), FILO (lane 4), ROM (lane 5), MV1 (lane 6), MV3 (lane 7), MV5 (lane 8), MV7 (lane 9), MV10 (lane 10). M, 100 bp DNA ladder (Thermo Fisher Scientific, Waltham, MA, USA).

Positive results were obtained for all EVOOs with the presence of a 290 bp amplicon, checked by means of a 2% agarose gel (Figure 1), corresponding to the *trn*L intron region as confirmed by successive direct sequencing using the same primers used for the initial genomic amplification.

### 2.3. PCR-Based Methods for Aspergillus Detection

After testing the amplifiability of the DNA from the oil samples, a new set of primers, IT1fw-IT1rv in Table 3, was designed to amplify a well-preserved part of the ITS1 region of the genome of *Aspergillus* spp. in order to ascertain the possible mold contamination of the EVOO samples. Our decision to use the ITS1 nuclear region as the target for the detection of this filamentous fungus was based on the previous identification of *Aspergillus flavus* as a specific fungal colony growing on olive fruit obtained from a Calabrian olive mill, as described in Section 4.

**Figure 2:**
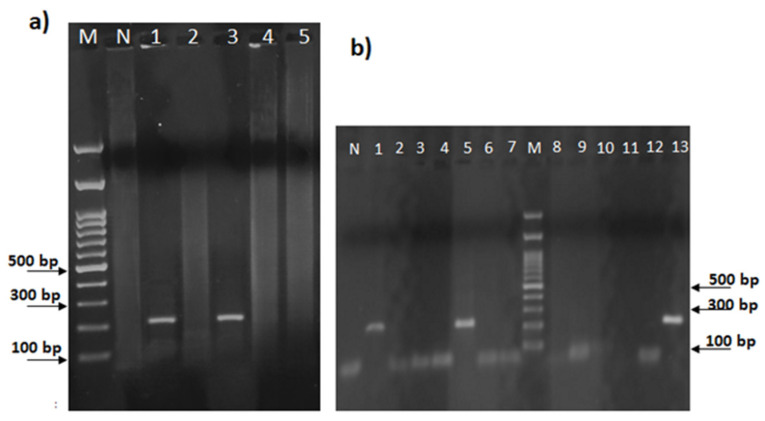
(**a**) Agarose gel electrophoresis of the ITS1 products obtained by PCR amplification of the genomic DNA from *Aspergillus flavus* (lane 1), *Olea europaea* (lane 2), *Aspergillus niger* (lane 3), *Listeria monocytogenes* (lane 4), and *Salmonella enterica* subsp. *enterica* sv. Typhimurium (lane 5); (**b**) Agarose gel electrophoresis of the ITS1 amplicon obtained by PCR amplification of the genomic DNA from *Aspergillus flavus* (lane 1) and EVOO representatives, namely CAS (lane 2), NOC (lane 3), PEN (lane 4), FID (lane 5), ROM (lane 6), FILA (lane 7), MV1 (lane 8), MV2 (lane 9), MV3 (lane 10), MV4 (lane 11), MV5 (lane 12), MV6 (lane 13). Lanes M and N represent 100 bp of the DNA ladder marker (Thermo Fisher Scientific, Waltham, MA, USA) and the negative control, respectively.

Therefore, aliquots (approximately 20 ng) of genomic DNA from two *Aspergillus* species, namely *Aspergillus flavus* and *Aspergillus niger*, from Carolea leaves (an *Olea europaea* variety), from a Gram-positive (*Listeria monocytogenes*) and a Gram-negative (*Salmonella enterica* subsp. *enterica* sv. Typhimurium) bacterium, and from the olive oil samples investigated here (Table 1), were subjected to an endpoint PCR assay using the IT1fw-IT1rv oligonucleotides (Table 3). Only for the two *Aspergillus* spp. and for two EVOOs, namely FID and MV6, was a single band of the expected size (about 220 bp) obtained, while none of the other samples tested showed any distinct amplification (Figure 2a,b).

**Figure 3:**
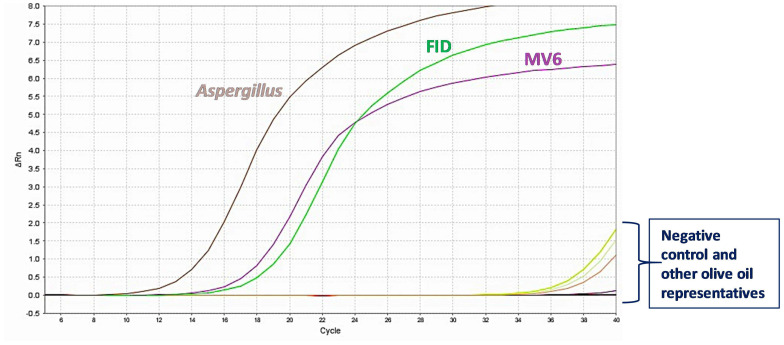
Real-time PCR amplification plot (ΔRn vs. cycle number) for the ITS1 region in *Aspergillus* spp. and some of the EVOO samples investigated in this study (Table 1). Curves denote successful amplification of positive samples.

RT-PCR is not only a faster method, but also more sensitive than conventional PCR. Therefore, we decided to validate the above results by performing a RT-PCR run using the ITS1 primers, 15 ng of each EVOO DNA template, and the SYBR^®^ fluorescent marker. An excerpt of the results is given in Figure 3.

Amplification curves, with Ct values ranging from 10 to 14, were again obtained for the FID and MV6 oil samples, respectively, with melting curves showing a single melting peak each and agarose gel electrophoresis indicating the absence of non-specific SYBR^®^ Green PCR products (Figure 3). No positive signal was evident when other EVOO DNA samples were used as templates (Figure 3).

**Figure 4:**
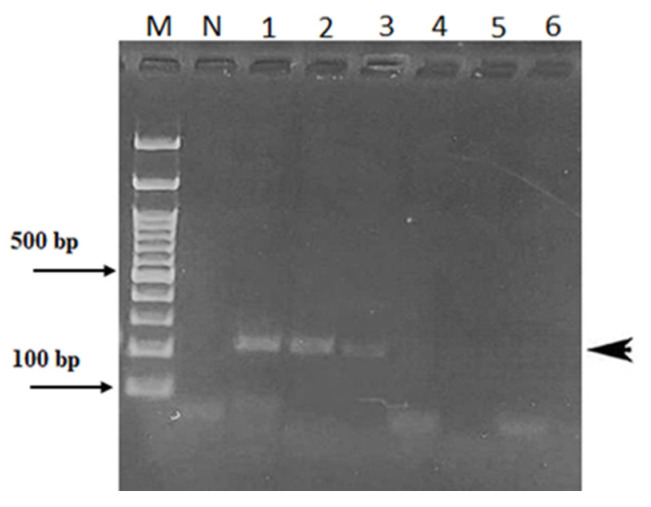
Agarose gel showing the limit of detection of the 220 bp amplicon (black arrowhead) after endpoint PCR with the primers IT1fw and IT1rv (Table 3) of different amounts of *Aspergillus flavus* DNA. Lane 1: 100 ng/μL; lane 2: 10 ng/μL; lane 3: 1.0 ng/μL; lane 4: 100 pg/μL; lane 5: 10 pg/μL; lane 6: 1.0 pg/μL; M: 100 bp Ladder (Thermo Fisher Scientific, Waltham, MA, USA); N: Negative.

The lowest amount of *Aspergillus* DNA template detected by both endpoint PCR and RT-PCR procedures was found to be approximately 1 ng (Figure 4). Although valid diagnostic tools, both of the above PCR-based assays are time-consuming procedures requiring costly and sophisticated equipment as well as highly trained and experienced personnel.

### 2.4. Primer Design, Specificity, and Sensitivity of the LAMP Assay

**Figure 5:**
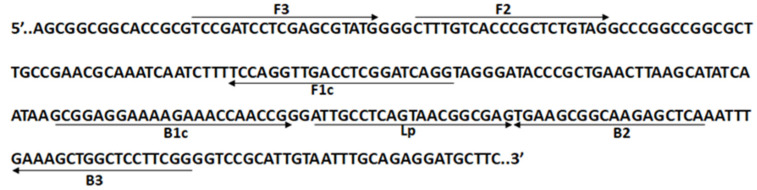
Annealing position and orientation of the LAMP primers, listed in Table 3, on the ITS2-28S sequence of *Aspergillus flavus* (GenBank MG745384).

The LAMP technique has been used for over a decade as a rapid, simple, and robust method for the detection of several phytopathogenic agents, including virus, bacteria, and fungi [24]. Therefore, in an attempt to reliably certify the quality of the EVOOs sampled here, we designed five specific LAMP primers based on the ITS2-28S sequence of *Aspergillus flavus* available in GenBank (MG745384) (Figure 5, Table 3).

**Figure 6:**
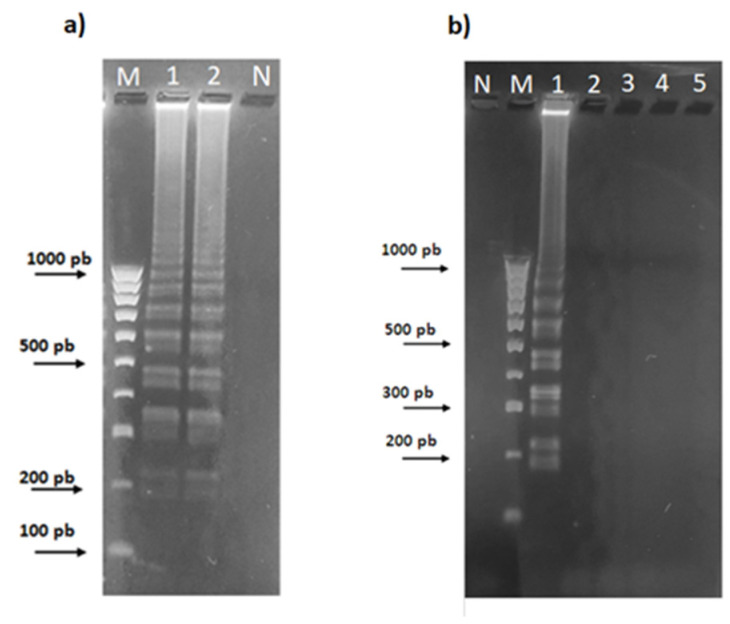
(**a**) Electrophoretic analysis of ITS2-28S LAMP products obtained after isothermal amplification of genomic DNA from *Aspergillus flavus* (lane 1) and *Aspergillus niger* (lane 2). Lanes N and M represent the negative control and 100 bp of the DNA ladder (Thermo Fisher Scientific, Waltham, MA, USA), respectively; (**b**) Primer specificity of the LAMP assay for the detection of *Aspergillus* spp. DNA. Lanes N and M represent the negative control and 100 bp of the DNA ladder (Thermo Fisher Scientific, Waltham, MA, USA), respectively. Lanes 1–5 represent different genomic DNAs: 1, *Aspergillus* spp.; 2, *Olea europaea*; 3, *Alternaria* spp.; 4, *Fusarium* spp.; 5, *Listeria* spp.

An initial amplification of *Aspergillus flavus* and *Aspergillus niger* genomic DNA was carried out in order to optimize the isothermal conditions to be applied. A LAMP product with a typical pattern of ladder-like DNA fragments on an electrophorezed agarose gel was observed for both *Aspergillus* DNA samples within 40 min of the start of the amplification reaction at 65 °C in a simple heating block (Figure 6a).

The novel LAMP primers showed no cross-reactivity with other species. Indeed, after the isothermal amplification of genomic DNA from *Olea europaea* and other ascomycete fungi and bacterial representatives, no LAMP products were obtained (Figure 6b).

These results demonstrated that the novel set of ITS2-28S LAMP primers developed here could be used specifically and rapidly in practical applications to detect *Aspergillus* spp. DNA.

**Figure 7:**
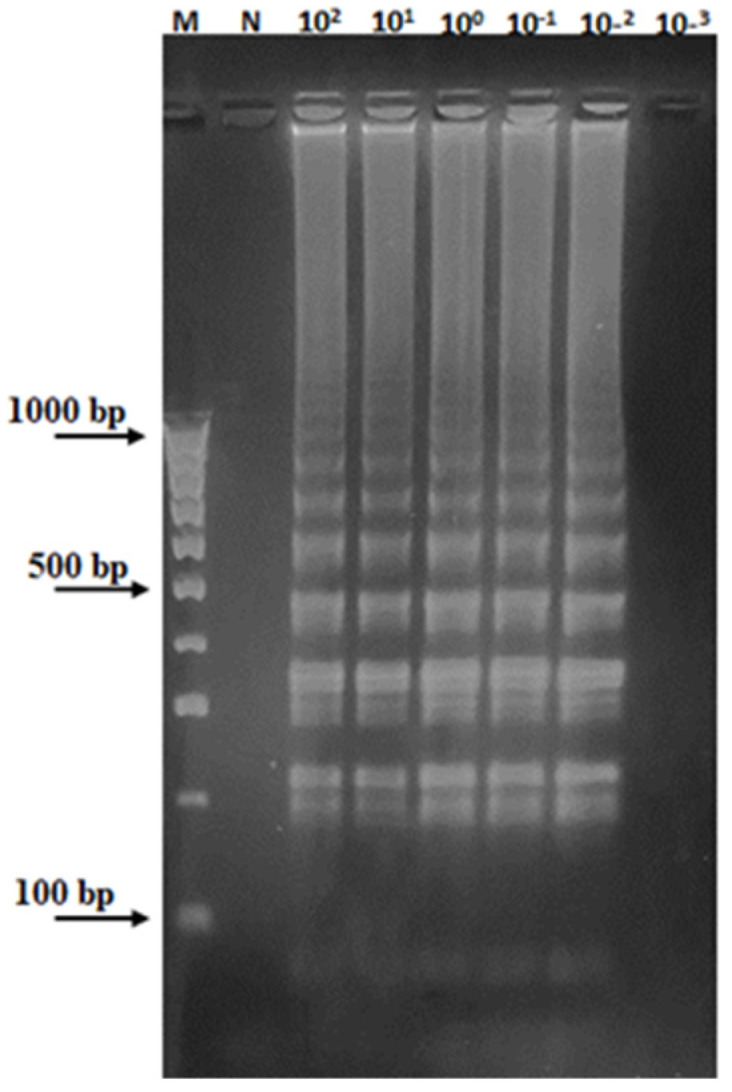
Sensitivity analysis of the LAMP assay for the detection of *Aspergillus* spp. DNA. Lanes M and N represent 100 bp of the DNA ladder marker (Thermo Fisher Scientific, Waltham, MA, USA) and the negative control, respectively. Different amounts of *Aspergillus* DNA, ranging from 100 ng·to 1 pg,·were added to the reaction mixtures to perform the LAMP.

Next, to evaluate the sensitivity of the LAMP assay, different amounts of extracted *Aspergillus* total DNA, ranging from 100 ng to 100 fg, were used as template DNA. The ladder-like DNA pattern was recovered when at least 10 pg of *Aspergillus* DNA was added to the reaction mixture (Figure 7). This means that the sensitivity of the LAMP was significantly higher than that observed for the endpoint PCR and the RT-PCR assays previously tested.

**Figure 8:**
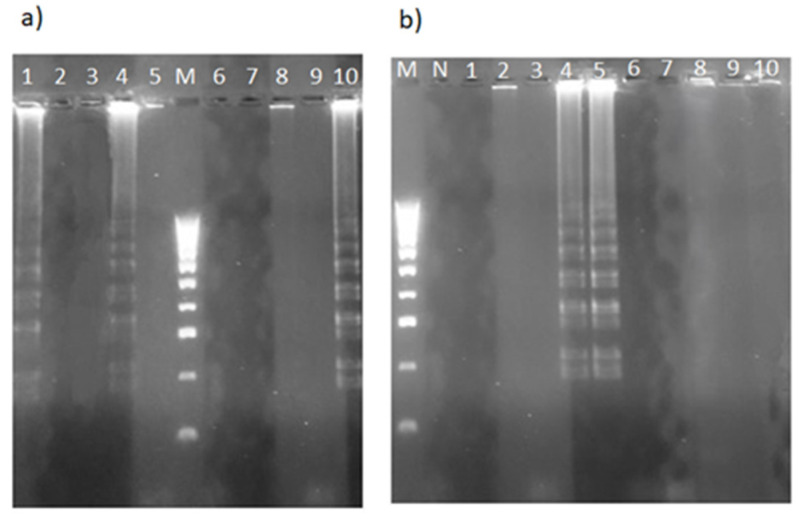
Electrophoretic analysis of LAMP products from monovarietal (**a**) and multivarietal (**b**) olive oils analyzed in this study (Table 1). Samples in (**a**): NOC (lane 1), CAS (lane 2), ROM (lane 3), FID (lane 4), FILO (lane 5), FILA (lane 6), CAR (lane 7), OTT (lane 8), CIC (lane 9), PEN (lane 10). Samples in (**b**): MV1 (lane 1), MV2 (lane 2), MV3 (lane 3), MV6 (lane 4), MV10 (lane 5), MV4 (lane 6), MV5 (lane 7), MV7 (lane 8), MV8 (lane 9), MV9 (lane 10). Lanes M and N represent 100 bp of the DNA ladder marker (Thermo Fisher Scientific, Waltham, MA, USA) and the negative control, respectively.

The next step was to use the LAMP primer set to detect *Aspergillus* DNA in EVOO samples. As shown in Figure 8a,b, three monovarietal (PEN, NOC, FID) and two multivarietal (MV6, MV10) olive oils with positive results in the LAMP test showed typical DNA ladder patterns when electrophoresed on an agarose gel.

However, it is known that this method of detecting the LAMP reaction may give false positive results due to contamination of the sample (i.e., when opening the reaction tube). In order to overcome this problem and obtain unambiguous results, a real-time LAMP assay (RT-LAMP) was carried out using a mini portable diagnostic instrument, the ICGene (Enbiotech Group s.r.l., Palermo, Italy). Due to the availability of specific diagnostic kits, this device has been successfully tested for the detection of a wide range of pathogens, especially viruses and bacteria.

Therefore, we wanted to use it for the validation of our novel LAMP method for the detection of *Aspergillus* spp. in the EVOO samples under investigation. For the RT-LAMP assay using the ICGene instrument, we used the typical LAMP reaction mixture but added an intercalating fluorescent dye (0.5 μL NEB 50× Fluorescent Dye), which allows the direct visualization of the results in a tablet with a specific application.

**Figure 9:**
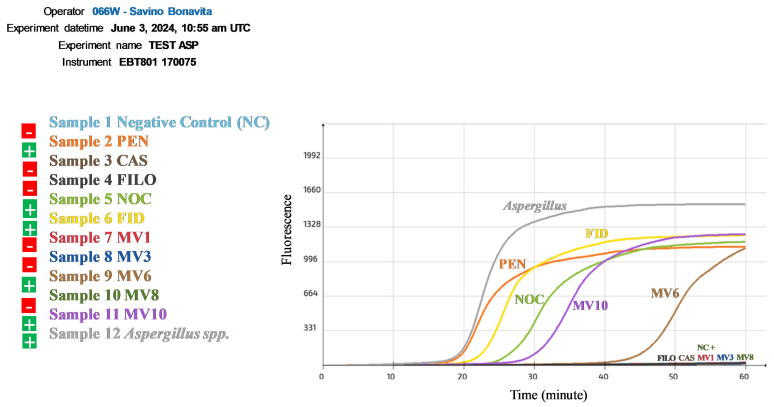
Amplification curves obtained from the ITS2-28S real-time LAMP assay in *Aspergillus* spp. and some EVOO samples analyzed in this study using the ICGene portable instrument. In this graph, the Y-axis shows the fluorescence absorbance during the reaction and the X-axis denotes the time in minutes taken for the reaction to start.

Interestingly, the RT-LAMP results agreed with the agarose gel electrophoresis of the LAMP products, since positive reactions were obtained for the same olive oil samples (PEN, NOC, FID, MV6, MV10), as illustrated in Figure 9 by the sigmoidal curves which have distinct initiation times.

## 3. Discussion

The overall quality of olive oils depends on their organoleptic characteristics, shelf life, nutritional value, and microbiological and toxicological safety, all of which are of great importance in the production and marketing of olive oils. To be considered a high-quality olive oil, it must meet the strict chemical criteria set by the IOC [4] in terms of free acidity, peroxide content, and other quality parameters.

Calabria is one of the most important olive-growing regions in Italy and the second-largest producer of olive oil in the country. This study started with the quality control of a number of Calabrian EVOOs, obtained from olive mills and supermarkets, using conventional chemical analyses. It was particularly noticeable that some samples of olive oil, both monovarietal and multivarietal, showed high levels of peroxides, which usually indicate a higher formation of primary oxidation products. In fact, oxygen is a key parameter of oil quality because it contributes to lipoxygenase reactions and, thus, to good organoleptic characteristics. For example, the peroxidase values of PEN, FID, and MV10 were greater than 30 meqO2/kg (Table 2), which is higher than the limit set by the IOC [4] for EVOO. The increase in the peroxide value of an olive oil is generally caused by several factors, such as the processing conditions (harvesting, transport, and storage of the olives) [33], but it is also often the result of pathogen contamination.

Olive oil is usually considered a food product that does not possess a suitable environment for the growth of various microbes. However, several studies have shown that even this “golden liquid” can be contaminated, particularly with certain species of mold fungi, such as *Aspergillus*. This can reduce the quality of the oil and its nutritional value and pose serious health risks to the consumer. Some *Aspergillus* species are, indeed, very dangerous, as they can produce mycotoxins under certain conditions, making food unfit to eat. It is therefore essential to develop efficient and accurate analytical methods to monitor and detect the contamination of olive oil in order to protect consumer health. Traditionally, the detection of fungal infections in foods has been based on morphological characteristics, which require long processing times and a well-equipped laboratory. Conversely, DNA-based methods using polymorphic chloroplast and/or nuclear sequences have been successfully used in recent decades for the rapid and accurate identification of fungal pathogens contaminating various food products [34,35,36]. However, the presence of contaminating molds in olive oil was rather difficult to detect, even considering the low recovery and, particularly, the low purity of the DNA obtained from this complex matrix. In fact, DNA extracted from olive oil may contain phenolic compounds or residual polysaccharides that may act as DNA polymerase inhibitors and may affect the quality of the subsequent PCR reaction by causing non-specific amplification [37,38].

Nevertheless, we were able to isolate sufficient DNA (≥5 ng/μL) from all EVOO samples, albeit of low quality (O.D.260/O.D.280 > 1.3), but still amplifiable and, later, successfully used that for the sequencing analysis. Our PCR-based assays, specifically endpoint and RT-PCR, both based on the ITS1 region of *Aspergillus* spp., confirmed the fungal infection of two of the sampled EVOOs, which were also among those with high peroxide levels (Table 2). Although reliable and sensitive, with comparable detection limits (1 ng) (Figure 4), both endpoint PCR and RT-PCR require expensive equipment and reagents, which limit their potential as rapid, inexpensive, field diagnostic tools. In contrast, the LAMP assay, which has been widely used to identify bacterial, viral, and fungal pathogens in plants and in food products [17,26,39], has many unique advantages. First, it generally needs only a small amount of DNA template. Secondly, because LAMP is an isothermal process, there is no need to raise or lower the temperature for nucleic acid amplification. This means no costly instrumentation is required to perform the above assay.

To the best of our knowledge, no studies have provided simple and rapid LAMP protocols for directly detecting fungal contaminants in olive oil. In this study, the strand-displacing activity of the *Bst* DNA polymerase and newly developed ITS2-28S LAMP primers were used to amplify DNA isolated from EVOO samples. The amplification yielded positive products in just 40 min, with a ladder-like pattern when verified by agarose gel electrophoresis. Notably, the LAMP assay identified more *Aspergillus*-contaminated EVOOs (5 out of 20), even at very low mold DNA concentrations, confirming that it is much more sensitive than endpoint PCR and RT-PCR assays (Figure 8a,b). In addition, the specificity of the LAMP assay reported here was demonstrated by the absence of amplification of non-target pathogens such as *Fusarium lateritium* and *Alternaria* spp., which are commonly found in olive leaves and oils [40]. False positives and non-specific binding are sometimes regarded as the main disadvantages of the LAMP method. Indeed, it should be noted that the methods in which the tubes are opened after amplification and the products are run on an agarose gel can lead to carry-over contamination and therefore to non-specific and false results. This can be avoided by the use of fluorescent dyes, so that fluorescent products can be detected by means of real-time amplification curves [41,42].

The RT-LAMP method, which was successfully carried out using a slight modification of our classic LAMP protocol in combination with the ICGene mini portable instrument, confirmed the results previously obtained (Figure 9). The different initiation times required for the DNA amplification of some oil samples, as reflected in our RT-LAMP results (Figure 9), can also be applied to the quantification of pathogen DNA content.

The use of a small, simple, and inexpensive device that can be used outside of a diagnostic laboratory, simply by connecting it to a tablet for displaying the results, was the major advantage of the implementation of the RT-LAMP on the ICGene. Although the latter method is promising, it will require further validation before a new specific *Aspergillus* detection kit can be developed and commercialized for use with this portable instrument.

## 4. Materials and Methods

### 4.1. Sample Collection

Monovarietal and multivarietal olive oils were collected from mills and supermarkets, respectively, from all five Calabrian provinces (Table 1). The names of the varieties from which the monovarietal olive oils were extracted have been abbreviated. All multivarietal samples were assigned acronyms to protect the identity of the manufacturer (Table 1).

### 4.2. Chemical Characterization of Oil Samples

Free acidity, the peroxide value, p-anisidine value, and spectrophotometric indices (K232, K270 and ΔK) were determined according to the analytical methods established by the IOC [4].

Particularly, the free acidity was determined as follows. A total of 45 mL of ethyl alcohol was added to 7.05 g of olive oil, followed by 50 µL of the chromogenic compound phenolphthalein. This solution was titrated with 0.025 M NaOH until the color changed slightly to light pink (AOCS Official Method Ca 5a-40). The free acidity is expressed as a percentage of oleic acid.

To determine the peroxide value, 30 mL of a solution of acetic acid and chloroform (3:2) and 0.5 mL of saturated KI were added to 5 g of olive oil. After mixing, 30 mL of water was added. The solution was titrated with 0.1 M sodium thiosulfate in the presence of a starch solution (1%, 1 mL) until the dark blue color disappeared (AOCS Official Method Cd 8-53). The peroxide value is expressed in mmole of peroxide (or active oxygen) per kg of oil (meqO2/kg).

The p-anisidine value (AnV) used to evaluate the secondary oxidation of oil or fat, mainly due to aldehydes and ketones, was determined according to the AOCS reference method Cd 18-90. Aldehydes in oil react with p-anisidine (0.25%) in glacial acetic acid for 10 min at room temperature, giving yellowish products that absorb at 350 nm.

The hydrolytic and oxidative parameters were also determined by using the CDR FoodLab^®^ system (http://www.cdr-mediared.com, accessed on 10 November 2023).

### 4.3. Oil Sample Preparation and Conventional PCR Assay

Total genomic DNA was extracted from 0.5 mL of all oil samples with the Olive Oil DNA Isolation Kit (Norgen Biotek Corporation, Thorold, ON, Canada). The extracted DNA was quantitated by NanoDrop 2000 spectrophotometer (Thermo Fisher Scientific, Waltham, MA, USA) and stored at −20 °C until use.

Amplifiability of the isolated oil DNA was assessed by conventional PCR of the chloroplast *trn*L intron using the primer combination *trn*LFw/*trn*LRev (Table 3). Primers were taken from Taberlet et al., 2007 [32]. PCR was set up for a 50 μL reaction mixture containing 50–100 ng of template DNA, 10.0 μL of 5X Wonder Taq Reaction buffer (including 5 mM dNTPs, 15 mM MgCl_2_), 2.0 μL of each of *trn*L forward and reverse primers (10 pm/μL) (Table 3), and 0.25–0.5 μL of Wonder Taq (1.25–2.5 U) (Euroclone S.p.A., Pero, Milano, Italy). The thermal cycling condition was 95 °C for 3 min followed by 30 cycles of 95 °C for 30 s, 55 °C for 30 s, 72 °C for 30 s, and a final extension at 72 °C for 5 min. The amplification reaction took place in an AB2720 Thermocycler (Applied Biosystems, Waltham, MA, USA). The resulting amplicon was purified directly from the PCR reaction mixture using the QIAquick PCR purification kit (Cat. No. 28104, Qiagen, Milano, Italy) and sequenced on both strands using the same primer pairs used in the PCR step by the Eurofins Genomics DNA sequencing service (https://www.eurofinsgenomics.eu, accessed on 13 October 2023).

### 4.4. Mold Isolation, Culture and Identification

Approximately 200 ripe olive fruits of the Grossa di Cassano (CAS) variety with internal mold growth were collected from a Calabrian olive mill in November 2022. The part of the olive fruit sample showing mold growth (0.5 cm in diameter piece) was cut off and transferred to a Petri dish containing Sabouraud dextrose agar medium. The plate was then incubated in a growth chamber at 25 °C in the dark for 7 days. One week later, the fungal colonies growing on the piece were identified as *Aspergillus flavus* under a 100× and 400× stereoscopic microscope and by PCR amplification and the subsequent sequencing of the ITS1 region using primers IT1fw-IT1rv (Table 3).

### 4.5. Aspergillus Flavus DNA Extraction

Approximately 200 mg of fresh *Aspergillus flavus* mycelia grown for 3 days at 30 °C in potato dextrose medium (20 g dextrose, 200 g potatoes infusion form, and 15 g agar, per 1 L water) and collected by filtration through Miracloth (Calbiochem, Germany) were used for genomic DNA isolation according to the procedure described by Tran et al. [43].

In addition, *Aspergillus niger* total DNA samples (Genebank accession numbers: MZ819922 and MZ819927) were kindly provided by Dr. Linda Bianco (ENEA Trisaia Research Centre, Matera, Italy).

### 4.6. LAMP Primers and Reaction

LAMP primers (outer primers, F3 and B3; inner primers, FIP and BIP; and a Loop primer, Lp) were designed based on the ITS2-28S sequence of *Aspergillus flavus*, obtained from GenBank (MG745384), using the commercial software of Primer Explorer V4 (http://primerexplorer.jp; Eiken Chemical Co., Ltd., Tokyo, Japan, accessed on 22 November 2023). Figure 5 and Table 3 show the positions and sequence, respectively, of the LAMP primers. The LAMP reactions were carried out in a reaction mixture containing 1× *Bst* DNA polymerization buffer, 8 U of *Bst* 2.0 WarmStart^®^ DNA Polymerase (New England BioLabs Inc., Ipswich, MA, USA), 0.2 μM each of F3 and B3 primers, 1.6 μM each of FIP and BIP primers, 0.4 μM of Loop primer, 0.2 mM dNTPs, and 100 ng of *Aspergillus* genomic DNA. Sterile deionized water was used as the template for the negative control. The mixtures were incubated for 30–60 min at 65 °C in a heating block (Thermomixer, Eppendorf, Milano, Italy) and the resulting LAMP product was detected by 2% agarose gel electrophoresis.

To assess the sensitivity of the LAMP method, a series of decimal dilutions of the *Aspergillus* DNA, ranging from 100 ng·μL^–1^ to 1 pg·μL^–1^, were prepared. The LAMP assay was also carried out combined with the ICGene device (Enbiotech Group S.r.l., Palermo, Italy), composed of a portable instrument and a real-time fluorometer, allowing the automatic interpretation of positive results by visualization of the amplification curve directly on the device display. Conversely, a linear or slightly oblique amplification curve indicated a negative result. All reactions were carried out three times, and the negative controls contained nuclease-free water.

## 5. Conclusions

The results of this study showed that all three DNA-based assays tested were effective, to varying degrees, in detecting *Aspergillus* contamination in the EVOO samples collected from the Calabrian provinces.

Although powerful and effective tools for mold detection, endpoint PCR and RT-PCR underestimated the real presence of *Aspergillus* spp. in EVOOs compared to the LAMP assay. Therefore, the high sensitivity, specificity, and rapidity of this latter method make it increasingly suitable to replace the laborious PCR-based assays in diagnostic laboratories for the assessment of mold contamination in olive oils.

In addition, the ability to perform a LAMP assay using ICGene allows for the early and rapid screening of olive oils directly at the production site. This may further ensure consumer safety and interests.

## Figures and Tables

**Table 1:** Monovarietal and multivarietal olive oils with the sampling area analyzed in this study.

Calabrian Province Manufacturing	Monovarietal Oil (Variety)	Multivarietal Oil
Vibo Valentia	Tonda Di Filogaso (FILO)	MV1
Vibo Valentia	Tonda Di Filadelfia (FILA)	MV2
Reggio Calabria	Ottobratica (OTT)	MV3
Reggio Calabria	Ciciarello (CIC)	MV4
Cosenza	Grossa Di Cassano (CAS)	MV5
Cosenza	Nocellara (NOC)	MV6
Crotone	Pennulara (PEN)	MV7
Crotone	Fidusa (FID)	MV8
Catanzaro	Carolea (CAR)	MV9
Catanzaro	Romanella (ROM)	MV10

**Table 2:** Most important quality parameters of EVOOs analyzed in this study.

	Free Acidity (% Oleic Acid)	Peroxide Value (meqO_2_/kg)	p-Anisidine Value (anV)
**Monovarietal oil**			
CAR	0.6	15.1	2.2
ROM	0.07	17.9	3.2
CAS	0.08	11.6	3.1
PEN	0.75	36.7	0.5
FID	0.63	32.4	3.2
CIC	0.12	18.3	1.6
OTT	0.23	13.9	2.0
NOC	0.73	27.7	1.2
FILO	0.24	18.5	2.1
FILA	0.51	20.4	1.4
**Multivarietal oil**			
MV1	0.2	16.4	4.6
MV2	0.25	19.5	0.5
MV3	0.22	13.7	4.9
MV4	0.59	16. 8	2.5
MV5	0.12	12.3	3.7
MV6	0.72	24.8	1.8
MV7	0.29	10.7	5.0
MV8	0.11	19.4	2.4
MV9	0.14	10.1	2.9
MV10	0.68	33.7	3.1

**Table 3:** Primer sequences for endpoint PCR and LAMP assays used in this study.

Assay	Primer	Sequence (5′–3′)
	*trn*LFw	CGAAATCGGTAGACGCTACG
Endpoint PCR	*trn*LRev	GGGGATAGAGGGACTTGAAC
	IT1fw-	GCGGAAGGATCATTACCGAG
	IT1rv	CAAGAGATCCATTGTTGAAAG
	F3	TCCGATCCTCGAGCGTATG
	B3	CCGAAGGAGCCAGCTTTC
LAMP	FIP (F2 + F1c)	CCTGATCCGAGGTCAACCTGGA- CTTTGTCACCCGCTCTGTAG
	BIP (B2 + B1c)	GCGGAGGAAAAGAAACCAACCG- TGAGCTCTTGCCGCTT
	Loop primer (Lp)	ATTGCCTCAGTAACGGCGAG

## Data Availability

Data are contained within the article.
